# Human mobility and urban malaria risk in the main transmission hotspot of Amazonian Brazil

**DOI:** 10.1371/journal.pone.0242357

**Published:** 2020-11-25

**Authors:** Igor C. Johansen, Priscila T. Rodrigues, Marcelo U. Ferreira

**Affiliations:** Department of Parasitology, Institute of Biomedical Sciences, University of São Paulo, São Paulo (SP), Brazil; Instituto Rene Rachou, BRAZIL

## Abstract

Malaria in the Amazon is often perceived as an exclusively rural disease, but transmission has been increasingly documented within and near urban centers. Here we explore patterns and causes of urban-to-rural mobility, which places travelers at risk of malaria in Mâncio Lima, the main malaria hotspot in northwestern Brazil. We also analyze rural-to-urban mobility caused by malaria treatment seeking, which poses an additional risk of infection to urban residents. We show that the rural localities most frequently visited by urban residents–typically farming settlements in the vicinity of the town–are those with the most intense malaria transmission and also the most frequent source localities of imported malaria cases diagnosed in the town. The most mobile urban residents are typically poor males 16 to 60-years old from multi-sited households who lack a formal job. Highly mobile residents represent a priority target for more intensive and effective malaria control interventions, that cannot be readily delivered to the entire community, in this and similar urbanized endemic settings across the Amazon.

## Introduction

Although the overall burden of malaria in Latin America and the Caribbean has decreased dramatically over the past two decades, transmission persists in 21 countries in the region, where 120 million people are estimated to be currently exposed to some risk of infection [1]. The Amazon Basin, a vast territory that extends over Bolivia, Brazil, Colombia, Ecuador, Guyana, French Guiana, Peru, Suriname, and Venezuela, contributes approximately 90% of the region’s malaria burden [2].

Malaria in the Amazon has traditionally been perceived as a disease affecting poor rural communities, with most reported infections acquired in remote riverine villages [3,4], frontier farming settlements [5,6], gold mining [7,8], and Amerindian reserves [9–11]. Indeed, malaria rates tend to be lower in cities and towns, compared to surrounding rural settings, due to multiple factors such as improved housing and access to healthcare and limited availability of mosquito vector habitats [12]. Nevertheless, since the mid-1990s malaria cases have been increasingly reported within and near urban centers in the Amazon, consistent with sustained transmission in or around towns across the region [13–20].

The Amazon Basin of Brazil is facing an accelerated urban growth–estimated at 2.4% per year between 2000 and 2010 [21] and characterized by massive rural-to-urban migration, unplanned housing, and inadequate infrastructure–that challenges its conventional representation as a densely forested territory interspersed by small and isolated human settlements [22]. Urban residents now account for 72.5% of the region′s population and large cities with >500,000 inhabitants are home to almost 20% of the 24.4 million Amazonians [21]. Urbanized spaces ranging from metropolitan areas to small towns sprawling into the rainforest gradually became more tightly articulated to the surrounding farming settlements, riverine villages, and even indigenous communities [23,24]. This process extends to rural spaces some socioeconomic and spatial relations that are typical of urban centers, blurring the traditional rural-urban boundary, and fosters human mobility across the rural-urban interface as a key component of new livelihood and income diversification strategies [25,26]. Rural families typically travel to the nearest town or city at least once a month to sell their crops, purchase goods, and receive social benefits from conditional cash transfer programs and rural retirement programs [27]. Conversely, newly arrived urban families often maintain both urban and rural residences and rely on agricultural production for subsistence or additional income [26–28]. These factors leading to increased human mobility are key drivers of urban malaria risk, as parasites from rural villages are introduced and may spread in densely populated and receptive urban spaces, potentially leading to explosive epidemics or sustained endemic propagation of parasites [16,20,29].

Malaria transmission rates in Brazil are nowadays greatest in the upper Juruá Valley, next to the border with Peru [30]. With <0.5% of the Amazon's population, the region contributes 18% of the overall country's malaria burden, estimated at 186,485 cases in 2018 [1]. Malaria transmission spreads to some urbanized spaces in the Juruá Valley region, where large population of anopheline mosquito vectors that thrive in locally abundant natural and artificial water habitats, mainly fish farming ponds, renders these areas receptive to malaria transmission [16,17,29,31].

Here we combine epidemiological surveillance data and travel histories to explore human mobility patterns in Mâncio Lima, the main urban malaria hotspot of Brazil. We found that the localities that are most frequently visited by urban residents are typically those with the most intense malaria transmission. These localities also contribute the vast majority of imported malaria infections diagnosed in the urban area. Importantly, source communities with more intense transmission, which are likely to drive most urban malaria risk, are not hard-to-reach riverine villages; instead, they are situated in the vicinity of the town, where effective public health interventions are easier to implement.

## Methods

### Study site

**Fig 1:**
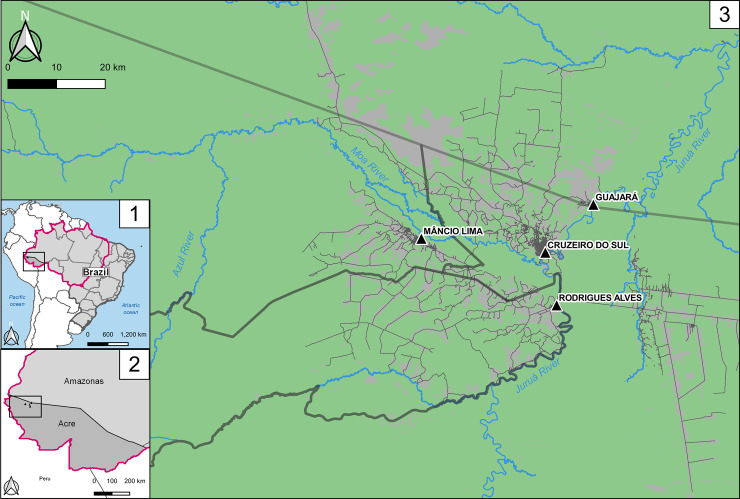
Location of the study site, the municipality of Mâncio Lima, Acre state, Brazil. 1: Brazilian Federal Units and the Amazon, also known as Legal Amazonia (magenta line in the map); 2: Acre and Amazonas states in dark and light gray, respectively, with the municipalities convered in this study highlighted in the weastern portion of the Fig 3: The municipalities of the upper Juruá Valley region: Mâncio Lima, Rodrigues Alves and Cruzeiro do Sul in Acre state and Guajará in Amazonas state. Dark thick lines represent the municipalities’ borders. The triangles show the town of each municipality, and in light green the forest cover in contrast with gray representing mostly deforested areas. Roads and streets are represented. Figure created with QGIS software version 3.14, an open source Geographic Information System (GIS) licensed under the GNU General Public License (https://bit.ly/2BSPB2F). Publicly available shape files provided from the Brazilian Institute of Geography and Statistics (IBGE) website (https://bit.ly/34gMq0S). Vegetated areas retrieved from Brazilian Institute for Space Research (2018) PRODES Project (https://bit.ly/33Q6wBD.) Roads and streets obtained from the Open Street Map Foundation website (https://bit.ly/36T2n1A). All utilized geographical data are under the Creative Commons Attribution License (CC BY 4.0).

The study site, the municipality of Mâncio Lima, covers a surface area of 5,453 km^2^ in the upper Juruá Valley region of Acre State, westernmost Brazil (Fig 1). Half of its 18,638 inhabitants reside in the town of Mâncio Lima, the only urban area in the municipality (07°36'51" S, 72°53'45" W). We note that urban areas in Brazil are defined according to relatively arbitrary administrative rules that do not necessarily consider population density and other internationally adopted criteria [32]. Here we delimitate the town of Mâncio Lima essentially as done by the Brazilian Institute of Geography and Statistics (IBGE) but extend the urban area to two urbanized neighborhoods (Iracema to the Northwest and Pé da Terra to the Southeast), which are situated along the main road that crosses the town, following the “urbanicity” criteria developed by Dal’Asta and colleagues [33] for use in this setting. These two neighborhoods had been originally classified as rural by IBGE.

Mâncio Lima has currently the highest annual parasite incidence (API; number of new laboratory-confirmed malaria cases per 1,000 population per year) for a municipality in Brazil, estimated at 422.8 in 2018 [34]. With a typical equatorial humid climate, the area receives most rainfall between November and April, but malaria transmission occurs year-round. Streams, wetlands rich in moriche palm trees, and natural and human-made fish farming ponds are widespread across the town of Mâncio Lima and serve as breeding habitats for malaria vectors [31]. The epidemiology of urban malaria in Mâncio Lima has been described in detail elsewhere [19].

### Sociodemographic data and travel histories

**Fig 2:**
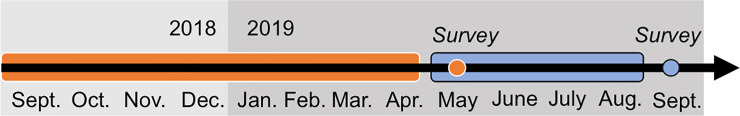
Timeline of the cross-sectional surveys of urban residents in Mâncio Lima, Brazil, during which mobility data were collected. In the May-June 2019 survey (orange circle in the figure), we collected information about overnight trips from September 2018 to April 2019; while in September-October 2019 survey (blue circle in the figure), we collected information about overnight trips from May to August 2019, then obtaining travel histories for the entire period of 12 months (September 2018 to August 2019).

A population census performed by our field team between November 2015 and April 2016 enumerated 9,124 permanent residents in the urban area of Mâncio Lima, with ages ranging between <1 month and 105 years (mean, 27.0; median, 22.0; SD, 20.0 years) and distributed into 2,329 households [19]. Our study sample comprises all members of randomly selected households that correspond to approximately 20% of all households enumerated and mapped during a census survey. Simple probability sampling was carried out using a list of households generated during the census survey to select 25% of all households in Mâncio Lima, allowing for empty houses and those we were unable to locate. Structured questionnaires applied to study participants were used to obtain sociodemographic data and travel histories. To this end, two consecutive cross-sectional surveys were carried out in the study site and targeted the same population sample (Fig 2). The first survey, between May and June 2019, comprised 2,015 subjects aged <1–105 years (mean, 28.2; median, 24.0; SD, 20.1 years) distributed into 522 households. The originally selected households were revisited during the second survey, between September and October 2019, which comprised 2,130 subjects aged <1–105 years (mean, 28.4; median, 24.5; SD, 20.1 years) distributed into 562 households. Demographic, socioeconomic and occupational/behavioral information was obtained. We note, however, that the population sample was dynamic such that residents joining the household were enrolled and residents leaving the household between the two cross-sectional surveys were withdrawn. Since this is an exploratory observational study was originally designed to evaluate several sociodemographic, clinical, and laboratory outcomes, including human mobility-related outcomes, in the same population, no formal sample size and power calculations were made. The study was not originally intended to have statistical power to detect small differences between comparison groups and across different exposure strata, which are unlikely to be of public health significance.

To measure the spatial mobility of urban residents, individual reports of overnight trips within the past 6 months were collected during two consecutive cross-sectional surveys carried out 6 months apart. Reports included the total duration of each trip and its destination, allowing us to calculate the total number of days each individual spent in each destination between September 2018 and August 2019. Only trips for which the destination, resolved at the locality level (as defined below), was situated in the upper Juruá Valley region were considered (77.1% of all overnight trips). Original anonymized data are available in S1 Dataset.

### Additional data sources

We retrieved all malaria case notifications from the upper Juruá Valley region (combined 2020 population estimate, 144,671 inhabitants) that were entered into the electronic malaria notification system of the Ministry of Health of Brazil between January 2016 and December 2018. Because malaria is a notifiable disease in Brazil and diagnostic testing and treatment are not available outside the network of government-run health care facilities, the database comprises the vast majority of laboratory-confirmed malaria episodes countrywide [35].

The upper Juruá Valley has been divided, for operational malaria control purposes, into smaller geographic units, or “localities”, with shared epidemiological and ecological characteristics [36]. The central points of the localities (typically a health post or school) were georeferenced using hand-held GPS receivers, all dwellings were identified and given a unique identifier, and all residents were enumerated during periodic census surveys carried out by the local malaria control program staff. We retrieved from the electronic malaria notification system the following locality-related information: (a) GPS coordinates, (b) population size, (c) number of locally acquired, laboratory-confirmed malaria episodes that were diagnosed and treated in the locality between 2016 and 2018, and (d) number of malaria episodes reportedly acquired in other localities that were diagnosed, treated and notified in the town of Mâncio Lima between 2016 and 2018 (i.e., *imported infections* [37]). An infection is routinely notified as imported when the patient reports overnight stays in an endemic site, within 15 days prior to diagnosis, which is different from the place where the infection has been diagnosed and notified [37].

From these data retrieved from the electronic malaria notification system we estimated the average API between 2016 and 2018 for every locality in the region and quantified rural-to-urban mobility caused by malaria-treatment seeking during this period, which determines the frequency of influx of infected individuals into the town (i.e., its *vulnerability* [37]). The numerator of API is the average number of locally acquired malaria cases between 2016 and 2018 and the denominator is the estimated population size for each locality in 2017 (based on the most recent census survey and adjusted for projections of population growth provided by the Brazilian Institute of Geography and Statistics). We note that the vast majority of imported infections in the study site are acquired in rural localities where malaria diagnosis facilities are not available, motivating treatment seeking in the town.

### Data analysis

Field-collected data, entered using tablets programed with REDCap [38], were cleaned and exported to Stata SE 15.0 (StataCorp, TX, USA) for statistical analysis. Proportions were compared with χ^2^ tests and correlations were investigated using the Pearson′s correlation test.

Multivariate regression models with either dichotomic or continuous outcome variables were run to identify correlates of urban-to-rural mobility. The dichotomic outcomes analyzed with logistic regression models were: (1) overnight trip outside the town within the past 12 months (no/yes) and (2) overnight trip to high-risk localities–i.e., those with an API greater than that of the town of Mâncio Lima (442 cases per 1,000 inhabitants)–within the past 12 months (no/yes). Given the nested structure of the data (individuals clustered into households), we used the “meqrlogit” STATA command to build mixed-effects logistic regression models that included the grouping variable “household” as a random factor. These models performed better than their Poisson counterparts, as judged by Akaike’s and Bayesian information criteria; therefore, only logistic regression results are presented here. The continuous outcome variables analyzed with negative binomial models were: (3) total number of overnights outside the town within the past 12 months and (4) total number of overnights in high-risk localities. Models were built with the “menbreg” STATA command, including the grouping variable “household” as random factor. For each outcome we ran two separate regression models: (a) one for the entire population and (b) one for economically active males (i.e. aged 16–60 years old), who tend to be more mobile due to occupational activities and behavior. For each analysis we present both adjusted and unadjusted coefficients.

Variable selection for the final models followed the hierarchical approach based on conceptual frameworks as suggested by Victora and colleagues [39]; hierarchical levels are shown in S1 Fig. Variables available were: age group (0–5; 6–15; 16–40; 41–60; 41–60; > 60 years); gender (female, male); literacy (illiterate; literate); terciles of a household wealth index (poorest; intermediate; least poor) that considers housing characteristics and assets, such as vehicles and home appliances, and was computed as described by Filmer and Pritchett [40]; if anyone in the household receives benefits from the federal *Bolsa Família* conditional cash transfer program [41] (no; yes); individual work status (does not work; formal employee; informal employee; employer); family head workstatus (does not work; formal employee; informal employee; employer); regular fishing (no/yes) and presence of a second residence outside the urban area (no, yes). All estimates are provided along with 95% confidence intervals.

### Ethics statement

The study protocol was approved by the Institutional Review Board of the Institute of Biomedical Sciences, University of São Paulo, Brazil, and by the National Committee of Ethics in Research (CONEP) of the Ministry of Health of Brazil (CAAE number 64767416.6.0000.5467). Written informed consent and assent were obtained from all study participants.

## Results

### Characteristics of the study population

Complete mobility information was available from 1,903 individuals, distributed into 504 households, who participated in both cross-sectional surveys. This total corresponds to 94.4% of individuals interviewed in May-June 2019 and 89.3% of those interviewed in September-October 2019. Sociodemographic characteristics of the study population are shown in S1 Table. Participants have a mean age of 28.3 years (range, <1–105 years; median, 24.0; standard deviation [SD], 20.2), with a male:female ratio of 0.95. Adults ≥25 years old reported 7.3 years of schooling on average, which corresponds to incomplete elementary school; 19.2% of study participants ≥15 years old are illiterate. Only 53.0% of individuals ≥18 years old reported to be currently working; of those, 8.6% are formally employed and the majority (55.7%) are informal employees who engage in seasonal farming in the surroundings of the town. Multi-sited households are common in Mâncio Lima– 16.8% (85 out of 504) of the families maintain both urban and rural residences. The 370 subjects living in multi-sited households were aged <1–92 years (mean, 27.3; median, 21.5; SD, 20.3), with a sex ratio close to 1:1 (186 females and 184 males). A total of 170 (45.9%) individuals live in households in the first (poorest) wealth index tercile. Moreover, 214 (57.8%) of the members of multi-sited households were directly of indirectly beneficiaries of the main conditional cash transfer program in Brazil (Bolsa Família), which targets the most socially vulnerable groups of the population. S2 Table provides the reported locations of second residences.

### Malaria rates in the town of Mâncio Lima and surrounding localities

**Fig 3:**
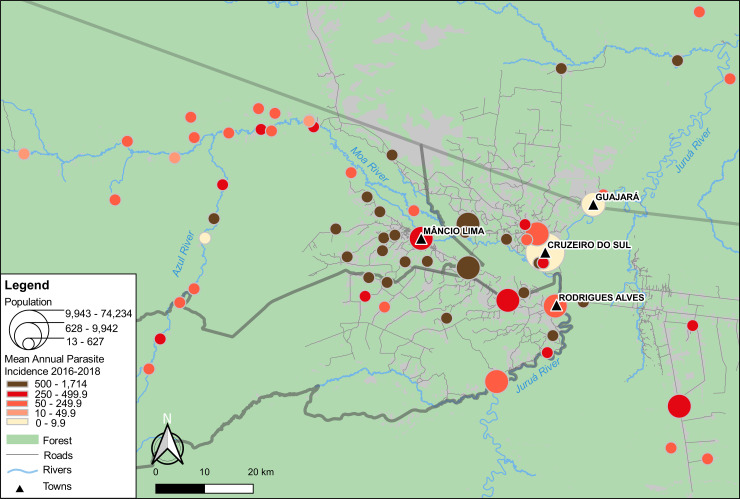
Map showing the location of the town of Mâncio Lima and the 65 localities in the upper Juruá Valley region that were mentioned as travel destinations by study participants. Georeferenced localities are represented by circles with size proportional to their population size and filled with tones from light yellow to dark brown that are proportional to malaria transmission intensity, using the average annual parasite incidences (APIs) for both *P*. *vivax* and *P*. *falciparum* between 2016 and 2018 as a proxy (higher APIs in darker tones).

Fig 3 shows that average APIs vary widely among the 65 localities in the upper Juruá Valley that were reported as destinations of overnight trips by urban residents in Mâncio Lima. The names of all destinations are provided in S2 Fig; separate IPA averages for *P*. *vivax* and *P*. *falciparum* are given in S3 and S4 Figs. Average API between 2016 and 2018 ranges across localities from 1 to 1,714 laboratory-confirmed malaria cases, regardless of the species, per 1,000 population per year; estimates for each locality are listed in S3 Table.

The average API for the town of Mâncio Lima during the same period was 442 cases per 1,000 population per year, which is surprisingly high for an urban setting. For comparison, the three other urban centers in the upper Juruá Valley region–namely, Cruzeiro do Sul (estimated urban population, 63,800 inhabitants), Rodrigues Alves (population, 13,200), and Guajará (population, 8,800), whose locations are indicated in Fig 3 –sustain substantially lower malaria transmission compared with Mâncio Lima, with mean APIs of 1, 50, and 8 malaria cases per 1,000 population between 2016 and 1018, respectively. Of note, three fourths of the 24 localities with very intense malaria transmission (API ≥ 500) are periurban farming settlements situated within a 20-km radius of the town of Mâncio Lima. Only three high-risk localities are remote riverine villages along the main local rivers (Juruá, Moa, and Azul).

Contrary to the commonly held perception of malaria as a disease of isolated communities deep in the rainforest in the Amazon, periurban agricultural settlements contribute most of the malaria burden in the upper Juruá Valley, what is especially apparent for the municipality of Mâncio Lima (Fig 3). Somewhat surprisingly, this pattern is even more marked for *P*. *falciparum* infections, which mostly cluster in the vicinity of the town, being much less frequent in more remote sites (S3 Fig). Locally transmitted malaria remains relatively infrequent in the towns of Rodrigues Alves and Guajará and the city of Cruzeiro do Sul (S4 Table).

### Urban-to-rural mobility and its determinants

Over one third (35.5%) of the study participants reported at least one overnight trip between September 2018 and August 2019, with a total of 34,150 overnights outside the town, corresponding to approximately 5% of their nights during the study period. Nearly half (47.0%) of the study participants who reported overnights outside the town were away for ≥1 month during the study period (one or more trips); 24.4% of them were away for ≥3 months.

**Fig 4:**
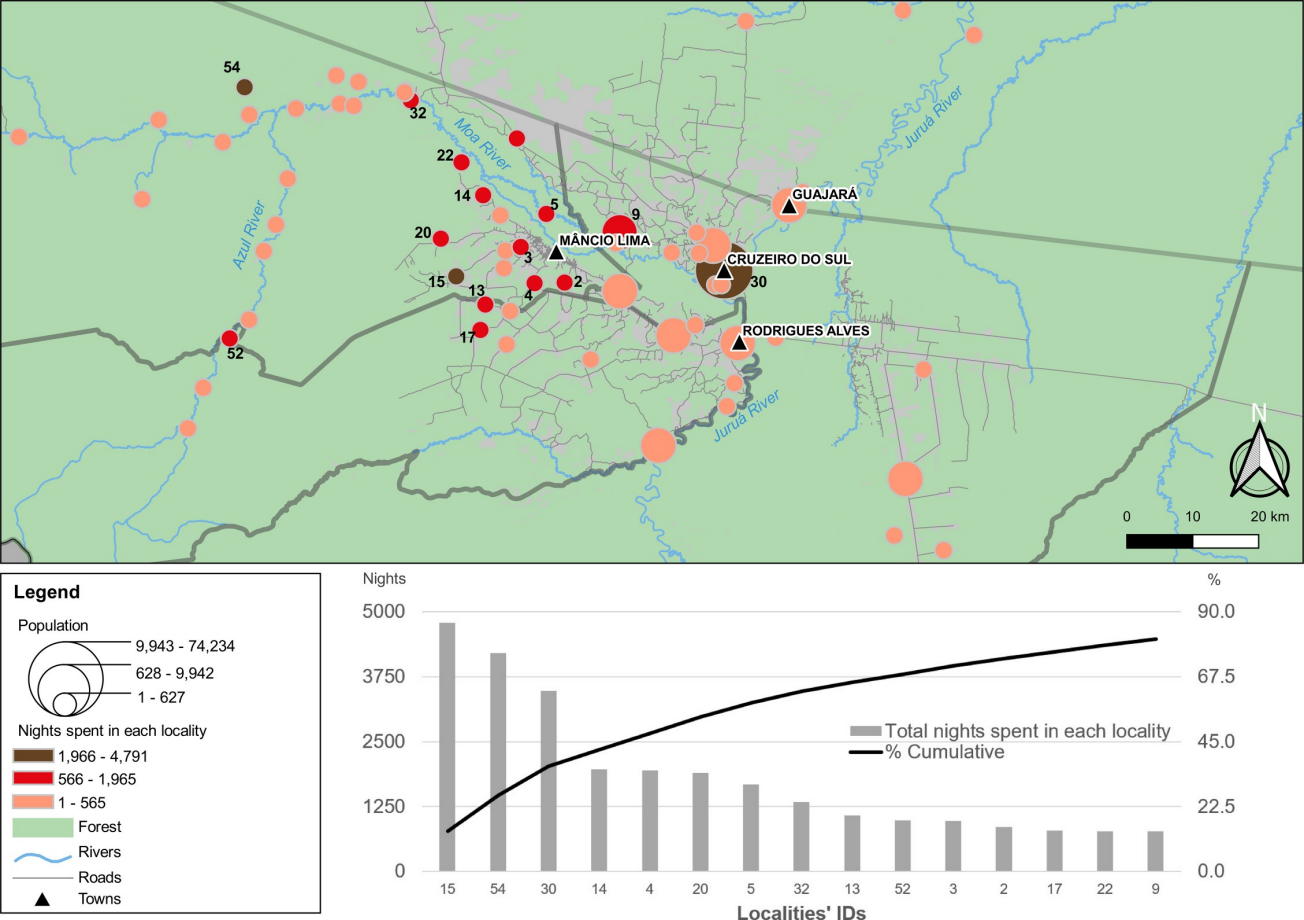
Trip destinations of residents in the town of Mâncio Lima according to number of overnight stays. As in Fig 1, the map shows the location of the town of Mâncio Lima and the 65 localities in the upper Juruá Valley region with overnigths reported by study participants. Georeferenced localities are represented by circles with size proportional to their population size and filled with tones from light orange to dark brown that are proportional to the number of overnights in each locality between September 2018 and August 2019 (larger number of overnights in darker tones). The lower panel shows the cumulative number and proportion of overnights in the top-15 localities; their identification codes (IDs) are shown in the map.

The three most frequent destinations are two small rural settlements (Tonico and Timbauba, #15 and #54 in Fig 4, respectively) and the nearest city (Cruzeiro do Sul, #30 in Fig 4). In contrast, the towns of Rodrigues Alves and Guajará attract relatively few visitors from Mâncio Lima. Tonico, the most visited locality, displays the highest API in the region, estimated at 1,714 cases per 1,000 inhabitants between 2016 and 2018 (S3 Table). Fifteen localities (14 of them rural) account for over 80% of overnights (Fig 4, lower panel; see also S5 Table). Importantly, the localities within 20 km of Mâncio Lima (32.3% of those shown in Fig 1) account for 58.1% of the total overnights outside the town. In addition, 48.7% of the reported overnights were in rural localities with API ≥ 500 (compare Figs 1 and 4), exposing urban visitors to substantial malaria risk. In other words, during the study period participants reportedly spent, on average, 2.4% of the their nights in high-risk localities, mostly in the vicinity of the town.

The number of overnights in each locality is not significantly correlated with its distance from the town of Mâncio Lima (Pearson´s *r* = -0.22, *P* = 0.09). We note, however, that the three most visited localities (Tonico, Cruzeiro do Sul and Timbauba) are outliers in the regression analysis, being disproportionately more visited than expected from their distance from the town of Mâncio Lima (S5 Fig). Because the city of Cruzeiro do Sul (4,204 overnights), situated at Euclidian distance of 26 km southeast from Mâncio Lima, is the largest urban center in the region, its attractiveness is easily understood. Moreover, as discussed below, Tonico and Timbauba are common sites for second residences of urban families. When these three outliers are removed from the regression analysis, a significant negative correlation is observed between the number of overnights and distance from the town (Pearson’s *r =* -0.36, *P =* 0.004), suggesting that study participants tend to spend more overnights in proximate localities, with the exceptions noted above.

Not surprisingly, members of multi-sited households display increased mobility. Indeed, 55.4% of study participants whose families have a second residence reported at least one overnight trip in the previous 12 months, compared with only 30.7% of those with no second residence (*P* < 0.01, χ^2^ test). The riverine village of Timbauba, along the Moa river (54 km northwest of the town in Euclidian distance), is the second most frequent site for a second residence and also the second most visited locality (4,204 overnights). Likewise, the farming settlement of Tonico (16 km west of the town by road), the most visited locality (4,791 overnights) and the highest API in the region, is among the top-five locations where study participants have a second residence.

Mixed-effects multiple logistic regression analysis identified five significant correlates of overall urban-to-rural mobility and four correlates of mobility to high-risk areas (Table 1). Common risk factors associated with both outcomes were: (a) male gender; (b) regular fishing; and (c) a second residence outside the urban area. Age bewteen 16 and 40 years was a statistically significant risk factor for overall mobility, while people older than 60 years old were less likely to spend their nights in high-risk areas. Informal employees were more likely to travel, but not necessarily to high-risk localities.

**Table 1:** Mixed-effects logistic regression analysis of correlates of urban-to-rural overall mobility (left columns) and mobility to high-risk areas (right columns) in the study population of Mâncio Lima, northwestern Brazil (n = 1,903).

	Overall mobility (overnight trip outside the town within the past 12 months)						Mobility to high-risk areas (overnight trip outside the town within the past 12 months to localities with an API greater than that of the town of Mâncio Lima)					
	Unadjusted model			Adjusted model			Unadjusted model			Adjusted model		
	OR^a^	(95% CI)^b^	*P*	OR^a^	(95% CI)^b^	*P*	OR^a^	(95% CI)^b^	*P*	OR^a^	(95% CI)^b^	*P*
Age												
0–5	Reference			Reference			Reference			Reference		
06–15	1.19	(0.7–2.0)	0.506	1.06	(0.6–1.8)	0.837	1.01	(0.5–2.0)	0.968	0.93	(0.5–1.9)	0.846
16–40	2.64	(1.6–4.2)	<0.0001	1.97	(1.2–3.3)	0.009	1.47	(0.8–2.7)	0.210	1.08	(0.5–2.1)	0.815
41–60	2.49	(1.4–4.4)	0.001	1.60	(0.8–3.0)	0.146	1.18	(0.6–2.5)	0.657	0.72	(0.3–1.7)	0.452
> 60	0.81	(0.4–1.6)	0.543	0.63	(0.3–1.3)	0.198	0.32	(0.1–0.9)	0.029	0.22	(0.1–0.6)	0.006
Gender												
Female	Reference			Reference			Reference			Reference		
Male	1.82	(1.4–2.4)	<0.0001	1.46	(1.1–2.0)	0.011	2.01	(1.4–2.9)	<0.0001	1.61	(1.1–2.4)	0.017
Work status												
Does not work	Reference			Reference			Reference			Reference		
Formal employee	1.38	(0.8–2.3)	0.198	0.90	(0.5–1.6)	0.709	1.38	(0.7–2.6)	0.312	1.15	(0.6–2.4)	0.695
Informal employee	3.13	(2.2–4.4)	<0.0001	1.61	(1.1–2.4)	0.022	2.21	(1.5–3.4)	<0.0001	1.44	0.8–2.5)	0.192
Employer	0.36	(0.0–4.2)	0.414	0.22	(0.0–2.6)	0.231	3.90	(0.1–105.5)	0.419	3.74	(0.1–108.8)	0.444
Wealth index^c^												
Poorest	Reference			Reference			Reference			Reference		
Intermediate	0.70	(0.4–1.2)	0.223	0.77	(0.43–1.4)	0.385	1.90	(0.8–4.4)	0.132	2.33	(1.0–5.5)	0.054
Least poor	0.76	(0.4–1.3)	0.336	0.75	(0.4–1.4)	0.368	1.29	(0.6–3.0)	0.554	1.35	(0.6–3.3)	0.515
Fishing												
No	Reference			Reference			Reference			Reference		
Yes	3.3	(2.3–4.7)	<0.0001	2.40	(1.6–3.5)	<0.0001	2.52	(1.58–4.0)	<0.0001	2.13	(1.3–3.6)	0.004
Second residence												
No	Reference			Reference			Reference			Reference		
Yes	5.05	(2.8–9.1)	<0.0001	5.59	(3.0–10.6)	<0.0001	6.8	(2.9–16.0)	<0.0001	7.58	(3.1–18.6)	<0.0001

^a^OR = odds ratio.

^b^CI = confidence interval.

^c^Wealth Index terciles.

Mixed-effects negative binomial regression models identified the following factors independently associated with increased number of overnights outside the town: (a) male gender, (b) age between 16 and 60 years, (c) informal employment, (d) regular fishing, and (e) second residence outside the urban area (Table 2). We note that the least poor study participants reported less overnights outside the town, compared with the lowest wealth stratum. The analysis limited to overnights in high-risk areas identified only two significant positive associations: (a) male gender and (b) second residence outside the town. Taken together, these results allow to identify high-mobility study participants as typically males aged 16 to 60 years old in the lowest wealth stratum, with informal jobs, who have a second residence outside the town and fish regularly. Further multivariate analyses limited to male participants aged between 16 and 60 years (n = 535), who comprise the main economically active segment of the population, essentially confirmed these findings (S6 and S7 Tables). Of note, individuals in the intermediate (but not highest) wealth stratum were found to be significantly less mobile in this curtailed dataset.

**Table 2:** Mixed-effects negative binomial regression analysis of correlates of urban-to-rural overall mobility (left columns) and mobility to high-risk areas (right columns), in the study population of Mâncio Lima, northwestern Brazil (n = 1,903).

	Overall mobility (total number of overnights outside the town within the past 12 months)						Mobility to high-risk areas (total number of overnights in localities with an API greater than that of the town of Mâncio Lima)					
	Unadjusted model			Adjusted model			Unadjusted model			Adjusted model		
	IRR^a^	(95% CI)^b^	*P*	IRR^a^	(95% CI)^b^	*P*	IRR^a^	(95% CI)^b^	*P*	IRR^a^	(95% CI)^b^	*P*
Age												
0–5	Reference			Reference			Reference			Reference		
06–15	1.31	(0.7–2.3)	0.362	1.24	(0.7–2.2)	0.463	1.01	(0.4–2.3)	0.990	0.91	(0.4–2.0)	0.821
16–40	3.52	(2.1–6.0)	<0.0001	2.73	(1.5–4.8)	0.001	2.06	(1.0–4.3)	0.052	1.61	(0.7–3.6)	0.243
41–60	4.14	(2.2–7.8)	<0.0001	2.42	(1.2–4.9)	0.015	1.75	(0.7–4.4)	0.237	1.06	(0.4–2.9)	0.911
> 60	1.27	(0.6–2.8)	0.566	0.97	(0.4–2.2)	0.938	0.72	(0.2–2.4)	0.595	0.48	(0.1–1.7)	0.256
Gender												
Female	Reference			Reference			Reference			Reference		
Male	2.43	(1.8–3.3)	<0.0001	1.94	(1.4–2.7)	<0.0001	3.04	(2.00–4.7)	<0.0001	2.76	(1.7–4.6)	<0.0001
Work status												
Does not work	Reference			Reference			Reference			Reference		
Formal employee	1.59	(0.9–2.8)	0.114	0.97	(0.5–1.8)	0.917	1.87	(0.8–4.6)	0.175	1.55	(0.6–4.0)	0.363
Informal employee	4.26	(2.9–6.2)	<0.0001	1.70	(1.0–2.8)	0.031	3.32	(1.9–5.7)	<0.0001	1.46	(0.7–3.0)	0.302
Employer	1.66	(0.1–22.0)	0.702	0.65	(0.0–9.4)	0.751	4.65	(0.2–130.1)	0.366	2.85	(0.1–102.2)	0.566
Wealth index^c^												
Poorest	Reference			Reference			Reference			Reference		
Intermediate	0.53	(0.3–1.0)	0.041	0.60	(0.3–1.1)	0.103	3.03	(1.0–9.4)	0.056	2.76	(1.0–7.8)	0.055
Least poor	0.53	(0.3–1.0)	0.047	0.51	(0.3–1.0)	0.037	1.74	(0.6–5.4)	0.335	1.14	(0.4–3.2)	0.805
Fishing												
No	Reference			Reference			Reference			Reference		
Yes	3.60	(2.4–5.4)	<0.0001	1.93	(1.3–3.0)	0.003	2.62	(1.4–4.9)	0.003	1.39	(0.7–2.7)	0.322
Second residence												
No	Reference			Reference			Reference			Reference		
Yes	13.3	(7.3–24.4)	<0.0001	15.46	(8.3–28.8)	<0.0001	60.18	(18.3–197.8)	<0.0001	60.99	(19.7–188.4)	<0.0001

^a^IRR = incidence rate ratio. Note that “incidence” here refers to the total number of overnights over 12 months.

^b^CI = confidence interval.

^c^Wealth Index terciles.

### Malaria treatment seeking and rural-to-urban mobility

Overall, 19,847 laboratory-confirmed malaria cases–including 16,347 *P*. *vivax* infections (82.4%) and 3,500 *P*. *falciparum* infections (17.6%)–were diagnosed, treated, and notified in the town of Mâncio Lima between January 2016 and December 2018. Of them, 5,389 (33.0%) *P*. *vivax* infections and 1,545 (44.1%) *P*. *falciparum* infections were classified as imported during routine case investigation (S8 Table). The ratio between locally transmitted and imported infections in the town is 2.0:1 for *P*. *vivax* and 1.3:1 for *P*. *falciparum*. These data indicate that a large number of subjects, either urban residents or not, who acquired malaria outside of the town regularly seek treatment in Mâncio Lima and expose their inhabitants to some risk of infection, given the malaria receptivity of the urban area.

**Fig 5:**
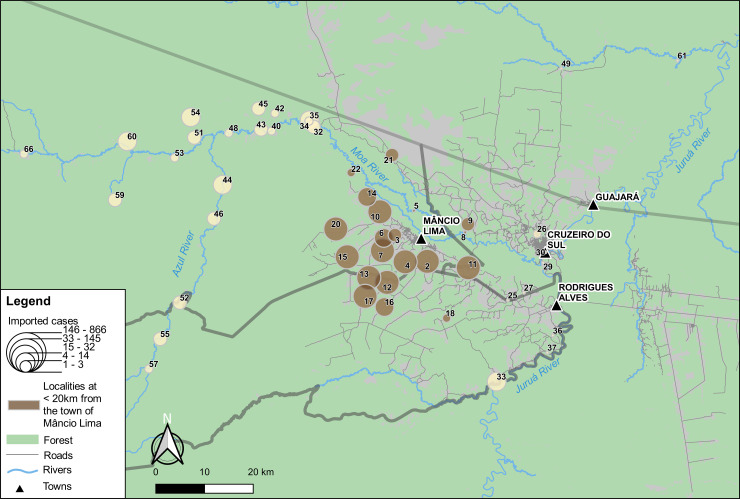
Map showing the localities recorded as source of imported malaria cases diagnosed and treated in Mâncio Lima between 2016 and 2018. Localities are represented by circles with size proportional to the total number of malaria cases acquired in each locality that were diagnosed and treated in the town of Mâncio Lima between 2016 and 2018 (quintiles). Localities situated at < 20km from the town (Euclidian distance) are shown in bown. The numbers of infections per locality that were imported to the town are shown in S8 Table.

We next investigated the geographic origin of imported malaria infections notified in Mâncio Lima. The vast majority of them (77.8% for *P*. *vivax* and 78.3% for *P*. *falciparum* infections) had one of the 65 localities shown in Fig 1 recorded as the most likely origin during routine case investigation (S8 Table). The most common putative source localities of imported cases are shown in Fig 5. Importantly, the top-10 source localities, none of them more than 20 km away from the urban area, account for 64.5% of *P*. *vivax* and 69.3% of *P*. *falciparum* infections imported into the town of Mâncio Lima. Permanent malaria diagnosis outposts are not available in these localities. Importantly, the number of imported cases correlates negatively with the distance between the putative source locality and the town (Pearson’s *r =* -0.45, *P =* 0.001).

## Discussion

The association between circular population movement and urban malaria risk has long been recognized in South America [42, 17] as well as in other endemic settings worldwide [e.g., 43–45]. Although public health policies cannot prevent directly human mobility, which is “driven mostly by need rather than choice” [46], they can proactively address some of the underlying causes and consequences of movements that contribute to malaria transmission.

Here we characterize patterns of spatial mobility in the main urban malaria hotspot of Brazil and their main determinants. We focus on the urban-to-rural mobility that places travelers at risk for malaria and rural-to-urban mobility caused by treatment seeking that poses a risk to urban residents, especially if rural visitors extend their stay in the town for selling crops, purchasing goods, or fully recovering from the current malaria episode. Importantly, the most common travel destinations are typically those with the most intense malaria transmission–essentially farming settlements situated within 20 km of Mâncio Lima, accessible to public health interventions, rather than remote riverine communities. These findings have clear implications for implementing effective malaria control policies in potential source communities that may fuel urban malaria transmission. The most mobile urban residents are poor males 16 to 60-years old from multi-sited households who lack a formal job in the town. Likewise, the most frequent source localities of imported cases diagnosed in Mâncio Lima are also situated within 20 km radius of the town.

The presence of multi-sited households in the Amazon [28] is part of a broader process called extended urbanization [23–25]. The traditional rural-urban divide in the region has been gradually replaced by a continuous gradient of “urbanicity”–typically urban features are extended to rural communities while towns and cities retain some “rurality” [33]. Our findings suggest that human mobility across the rural-urban gradient, mostly motivated by subsistence or commercial farming in peri-urban settlements, poses a continuous risk of malaria introduction into more urbanized and densely populated spaces.

These results can inform public health responses to prevent mobility-related urban and peri-urban malaria transmission across the Amazon, including large cities such as Manaus [18] and Porto Velho [15] in Brazil and Iquitos in Peru [14]. The first challenge consists in identifying the most mobile population strata who may contribute a large proportion of infections in the community [19]. Once identified, mobile individuals may be targeted with more intensive and effective interventions that cannot be readily delivered to the entire community. Importantly, the high-risk individuals in Mâncio Lima will acquire clinical immunity faster, after repeated infections [47], and eventually constitute a large clinically silent reservoir that carries malaria parasites across the rural-urban interface. Delivering personal protection measures, such as bed nets, and adequate access to diagnosis and treatment are examples of strategies to mitigate mobility-associated malaria risk.

Second, human mobility fuels malaria transmission in urban centers in the Amazon that are *receptive*–i.e., whose environmental conditions allow for malaria transmission from a human through a vector mosquito to another human [37]. Indeed, molecular analyses of parasite isolates provide evidence for sustained malaria transmission in the town of Mâncio Lima [20]. Vectors are increasingly abundant in this and other urbanized spaces in the Amazon and can sustain local malaria transmission. Typical larval habitats are natural water bodies in unplanned peri-urban settlements adjacent to forested areas [22] and natural and human-made fish farming ponds, now widespread in towns and cities across the region [16,29,31,48–50]. Extensive deforestation and environmental degradation may further displace vectors to more urbanized areas where suitable larval habitats are found [29]. Interestingly, highly productive larval habitants have also been increasingly found in areas dedicated to urban farming in tropical Africa (e.g., [51]). Larval source management with biological larvicides represents a logical approach to malaria control in urbanized spaces where breeding sites are relatively few, easy to find and readily accessible. It has been successful in African cities [52,53] and can drastically reduce anopheline larval density in fish farming ponds in the Amazon [54].

Third, imported infections diagnosed in urban communities across the Amazon do not necessarily originate in hard-to-reach traditional settlements. Instead, our results indicate that they are mostly acquired in farming settlements in the vicinity of the city or town, where farmers usually sell their crops and purchase goods. These findings imply that more intensive control interventions and better infrastructure for laboratory diagnosis and prompt treatment in nearby rural localities might drastically reduce the number of cases imported into the urban area. Moreover, as malaria transmission has been clearly linked to deforestation [29], environmental policies are also likely to contribute to malaria control in this and similar settings across the Amazon.

This study has some limitations. One is the possible recall bias in travel histories obtained at approximately six-month intervals, given the long time elapsed between the event and the interview. Alternatives to travel histories include the use of mobile phone data, which have been increasingly used to track human movements over time [55]. To this end, individuals are assigned to a primary cell phone tower based on the most frequently used tower at night. Travel to another tower catchment area is inferred if their location is recorded at that tower for more than one night. However, this strategy depends on relatively good mobile phone coverage, typically found in cities and towns [56] but absent in most rural sites in the Amazon. Recall bias may also affect the inference of the most likely site of infection, which was also self-reported, in the investigation of imported malaria cases. Finally, the use of routine malaria surveillance data, which are limited to clinical cases detected by conventional diagnostic methods such as microscopy or rapid diagnostic tests, is also a limitation. Routine surveillance typically overlooks chronic asymptomatic carriers of submicroscopic parasitemias who may seed infections in urban spaces over extended periods of time [57].

Despite these potential study limitations, we show that surveillance data combined with additional sociodemographic and mobility information from population-based surveys provides valuable information that can be explored for evidence-based planning and deployment of interventions aimed to reduce urban malaria risk across the Amazon.

## Supporting information

S1 Checklist STROBE checklist. (DOC)

S1 File Questionnaires used to collect mobility data during cross-sectional surveys. (PDF)

S1 Dataset Excel file containing the original anonymized mobility data used in our analyses. (XLSX)

S1 Table Sociodemographic characteristics of the study population. (DOCX)

S2 Table Sites for second residences of the residents in the town of Mâncio Lima. (DOCX)

S3 Table Per-locality Annual Parasite Incidence (API) in the municipality of Mâncio Lima and surrounding areas, 2016–2018. (DOCX)

S4 Table Malaria cases according to most likely local of infection (either urban or rural) for all municipalities in the Amazon and selected municipalities in the upper Juruá Valley of Brazil, 2016–2018. (DOCX)

S5 Table Per-locality number of overnight stays by Mâncio Lima urban residents, from September 2018 to August 2019. (DOCX)

S6 Table Mixed-effects logistic regression results with determinants of urban-to-rural overall mobility (left columns) and mobility to high-risk areas (right columns), for males 16–60 years old (n = 535). (DOCX)

S7 Table Mixed-effects negative binomial regression results with determinants of urban-to-rural overall mobility (left columns) and mobility to high-risk areas (right columns), for males 16–60 years old (n = 535). (DOCX)

S8 Table Most likely localities of origin of imported infections diagnosed and treated in the town of Mâncio Lima, 2016–2018. (DOCX)

S1 Fig Hierarchical causality model used for variable selection for multivariable models. (TIFF)

S2 Fig Localities surrounding the town of Mâncio Lima reported as travel destination by urban residents. (TIF)

S3 Fig *P*. *vivax* average annual parasite incidences (APIs) between 2016 and 2018 for the town of Mâncio Lima and the 65 localities in the upper Juruá Valley that were mentioned as travel destinations by study participants. Georeferenced localities are represented by circles with size proportional to their population size and filled with tones from light yellow to dark brown that are proportional to malaria transmission intensity, using the APIs as a proxy (higher APIs in darker tones).
(TIF)

S4 Fig *P*. *falciparum* average annual parasite incidences (APIs) between 2016 and 2018 for the town of Mâncio Lima and the 65 localities in the upper Juruá Valley that were mentioned as travel destinations by study participants. Georeferenced localities are represented by circles with size proportional to their population size and filled with tones from light yellow to dark brown that are proportional to malaria transmission intensity, using the APIs as a proxy (higher APIs in darker tones).
(TIF)

S5 Fig Correlation between the number of overnights of urban residents in Mâncio Lima in other localities in the upper Juruá Valley region and their distance in km from the town of Mâncio Lima. (TIFF)
