# Supplementary material for: Human mobility and urban malaria risk in the main transmission hotspot of Amazonian Brazil
Source: PLoS One. 2020 Nov 25;15(11):e0242357. doi: 10.1371/journal.pone.0242357 (PMC7688137; doi:10.1371/journal.pone.0242357)
Supplement: S1 File — (PDF) [file pone.0242357.s002.pdf]

III. EXPOSIÇÃO

ID	42. Nos últimos 8 meses dormiu fora da cidade de Mâncio Lima? *Não: 0 (Siga Q44)* *Sim: 1 (Siga Q42.1)* *Não sabe/ Não respondeu: 9 (Siga Q 44)*	Quantos dias em cada mês?							
		42.1 Set (30 dias)	42.2 Out (31 dias)	42.3 Nov (30 dias)	42.4 Dez (31 dias)	42.5 Jan (31 dias)	42.6 Fev (28 dias)	42.7 Mar (31 dias)	42.8 Abril (30 dias)

Códigos
Q43.01, Q43.11, Q43.21
Localidade:
0 - Rio Moa – onde?
1 - Rio Japiim – onde?
2 - Rio Azul – onde?
3 - Ramal do 20
4 - Ramal do Batoque
5 - Ramal do Feijão Insosso
6 - Ramal do Banho
7 - Igarapé Branco
8 - Igarapé da Bahia
9 - Ramal do Chaparral
10 - Timbauba
11 - Ramal dos Caetanos
12 - UFAC
13 - Ramal do Viola
14 - Área urbana de Cruzeiro do Sul
15 - Paraná do Pentecostes
16 - Ramal São Domingos
17 - Pafé do Havaí
18 - Aldeia Puyanawa
19 - Ramal do Tiririca
20 - Ramal do Generoso
21 - Polo Agroflorestal
77 - Outro local – onde?
99 - Não sabe/ Não respondeu

ID	43. MOBILIDADE - Locais mais visitados														
	43.0 Local 0					43.1 Local 1					43.2 Local 2				
	43.01 Localidade *Ver códigos* *Se 0 - Moa , 1 - Japiim, 2- Rio Azul ou 77 - Outro local: indique onde. E siga Q43.02 Se outro código, siga Q43.05*	43.02 Mun.	43.03 UF	43.04 *Urb: 0* *Rur: 1*	43.05 Total dias fora	43.11 Localidade *Ver códigos* *Se 0 - Moa , 1 - Japiim, 2- Rio Azul ou 77 - Outro local: indique onde. E siga Q43.12 Se outro código, siga Q43.15*	43.12 Mun.	43.13 UF	43.14 *Urb: 0* *Rur: 1*	43.15 Total dias fora	43.21 Localidade *Ver códigos* *Se 0 - Moa , 1 - Japiim, 2- Rio Azul ou 77 - Outro local: indique onde. E siga Q43.22 Se outro código, siga Q43.25*	43.22 Mun.	43.23 UF	43.24 *Urb: 0* *Rur: 1*	43.25 Total dias fora

III. EXPOSIÇÃO

ID	42. Nos últimos 4 meses dormiu fora da cidade de Mâncio Lima?<br>*Não: 0 (Siga Q44)*<br>*Sim: 1 (Siga Q42.1)*<br>*Não sabe/ Não respondeu: 9 (Siga Q44)*	Quantos dias em cada mês? 42.1 Maio (31 dias)	42.2 Jun. (30 dias)	42.3 Jul. (31 dias)	42.4 Ago. (31 dias)

Códigos

Q43.01, Q43.11, Q43.21 Localidade:

0 - Rio Moa – onde?
1 - Rio Japiim – onde?
2 - Rio Azul – onde?
3 - Ramal do 20
4 - Ramal do Batoque
5 - Ramal do Feijão Insosso
6 - Ramal do Banho
7 - Igarapé Branco
8 - Igarapé da Bahia
9 - Ramal do Chaparral
10 - Timbauba
11 - Ramal dos Caetanos
12 - UFAC
13 - Ramal do Viola
14 - Área urbana de Cruzeiro do Sul
15 - Paraná do Pentecostes
16 - Ramal São Domingos
17 - Pafé do Havaí
18 - Aldeia Puyanawa
19 - Ramal do Tiririca
20 - Ramal do Generoso
21 - Polo Agroflorestal
77 - Outro local – onde?
99 - Não sabe/ Não respondeu

ID	43. MOBILIDADE - Locais mais visitados									
	**43.0 Local 0**					**43.1 Local 1**				
	43.01 Localidade<br>*Ver códigos*<br>*(Se 0 - Moa, 1 - Japiim, 2- Rio Azul ou 77 - Outro local: indique onde e siga Q43.02. Se outro código, siga Q43.05)*	43.02 Mun.	43.03 UF	43.04<br>*Urb: 0*<br>*Rur: 1*	43.05 Total dias fora	43.11 Localidade<br>*Ver códigos*<br>*(Se 0 - Moa, 1 - Japiim, 2- Rio Azul ou 77 - Outro local: indique onde e siga Q43.12. Se outro código, siga Q43.15)*	43.12 Mun.	43.13 UF	43.14<br>*Urb: 0*<br>*Rur: 1*	43.15 Total dias fora
