# Supplementary material for: Human mobility and urban malaria risk in the main transmission hotspot of Amazonian Brazil
Source: PLoS One. 2020 Nov 25;15(11):e0242357. doi: 10.1371/journal.pone.0242357 (PMC7688137; doi:10.1371/journal.pone.0242357)
Supplement: S1 Table — (DOCX) [file pone.0242357.s004.docx]

S1 Table. Sociodemographic characteristics of the study population.

|  | **Variable** | **Categories** | **n (%)** |
| --- | --- | --- | --- |
| Demographic characteristics | Age | 0-5 | 210 (11.04) |
|  |  | 6-15 | 414 (21.76) |
|  |  | 16-40 | 801 (42.09) |
|  |  | 41-60 | 307 (16.13) |
|  |  | >60 | 171 (8.99) |
|  |  | Total | 1,903 (100) |
|  | Gender | Female | 977 (51.34) |
|  |  | Male | 926 (48.66) |
|  |  | Total | 1,903 (100) |
|  | Literacy | Illiterate | 538 (28.27) |
|  |  | Literate | 1,354 (71.15) |
|  |  | Did not answer | 11 (0.58) |
|  |  | Total | 1,903 (100) |
| Socioeconomic determinants | Wealth index (terciles)^a^ | Poorest | 791 (41.57) |
|  |  | Intermediate | 584 (30.69) |
|  |  | Least poor | 528 (27.75) |
|  |  | Total | 1,903 (100) |
|  | Social benefits from conditional cash transfer programs | No | 784 (41.20) |
|  |  | Yes | 1,119 (58.80) |
|  |  | Total | 1,903 (100) |
| Occupational and behavioral determinants | Individual work status | Does not work | 1,274 (66.95) |
|  |  | Formal employee | 177 (9.30) |
|  |  | Informal employee | 443 (23.28) |
|  |  | Employer | 7 (0.37) |
|  |  | Did not answer | 2 (0.11) |
|  |  | Total | 1,903 (100) |
|  | Family head work status | Does not work | 251 (13.19) |
|  |  | Formal employee | 416 (21.86) |
|  |  | Informal employee | 1,215 (63.85) |
|  |  | Employer | 16 (0.84) |
|  |  | Did not answer | 5 (0.26) |
|  |  | Total | 1,903 (100) |
|  | Regular fishing | No | 1,495 (78.56) |
|  |  | Yes | 384 (20.18) |
|  |  | Did not answer | 24 (1.26) |
|  |  | Total | 1,903 (100) |
|  | Second residence outside the town | No | 1,533 (80.56) |
|  |  | Yes | 370 (19.44) |
|  |  | Total | 1,903 (100) |

^a^ The number of individuals in each wealth index tercile is not equal because the average number of household members vary across terciles (greater in the poorest households).
