# Supplementary material for: Human mobility and urban malaria risk in the main transmission hotspot of Amazonian Brazil
Source: PLoS One. 2020 Nov 25;15(11):e0242357. doi: 10.1371/journal.pone.0242357 (PMC7688137; doi:10.1371/journal.pone.0242357)
Supplement: S2 Table — (DOCX) [file pone.0242357.s005.docx]

S2 Table. Sites for second residences of the urban residents in the town of Mâncio Lima.

| **ID** | **Locality name** | **Municipality** | **Second residences** |
| --- | --- | --- | --- |
| - | Missing information | - | 18 |
| 20 | COLONIA DO VINTE | Mancio Lima | 9 |
| 54 | C. TIMBAUBA - PDS SAO SAVADOR | Mancio Lima | 7 |
| 7 | BRASILIA | Mancio Lima | 6 |
| 15 | TONICO | Mancio Lima | 6 |
| 17 | HAVAI | Rodrigues Alves | 5 |
| 10 | SAO DOMINGOS | Mancio Lima | 4 |
| 13 | BAHIA | Rodrigues Alves | 4 |
| 32 | BELO MONTE | Mancio Lima | 3 |
| 52 | BOM SOSSEGO | Mancio Lima | 3 |
| 4 | BATOQUE | Mancio Lima | 3 |
| 30 | URBAN AREA | Cruzeiro do Sul | 2 |
| 14 | ALDEIA BARAO | Mancio Lima | 2 |
| 65 | RAMAL 11 | Cruzeiro do Sul | 1 |
| 25 | SAO PEDRO | Cruzeiro do Sul | 1 |
| 5 | AURORA | Mancio Lima | 1 |
| 59 | NOVO RECREIO | Mancio Lima | 1 |
| 66 | SERRA DO MOA F/P | Mancio Lima | 1 |
| 2 | JOSE BERNARDO | Mancio Lima | 1 |
| 45 | C. SAO PEDRO - PDS SAO SALVADOR | Mancio Lima | 1 |
| 43 | C. SAO FRANCISCO - PDS SAO SALVADOR | Mancio Lima | 1 |
| 44 | TRES UNIDOS | Mancio Lima | 1 |
| 46 | BURITI | Mancio Lima | 1 |
| 22 | BOM JARDIM | Mancio Lima | 1 |
| 3 | CARDOSO | Mancio Lima | 1 |
| 33 | PARANA DOS MOURAS | Rodrigues Alves | 1 |
