# Supplementary material for: Human mobility and urban malaria risk in the main transmission hotspot of Amazonian Brazil
Source: PLoS One. 2020 Nov 25;15(11):e0242357. doi: 10.1371/journal.pone.0242357 (PMC7688137; doi:10.1371/journal.pone.0242357)
Supplement: S3 Table — (DOCX) [file pone.0242357.s006.docx]

S3 Table. Per-locality Annual Parasite Incidence (API) in the municipality of Mâncio Lima and surrounding areas, 2016-2018.

| ID | Name | Municipality | Code (SIVEP) | API Total |
| --- | --- | --- | --- | --- |
| 15 | TONICO | Mancio Lima | 120033_15 | 1,714 |
| 21 | PARANA DO PENTECOSTE | Mancio Lima | 120033_52 | 1,597 |
| 61 | BADEJO DO MEIO (ESTR. DO GAMA) | Guajara | 130165_45 | 1,549 |
| 7 | BRASILIA | Mancio Lima | 120033_14 | 1,407 |
| 4 | BATOQUE | Mancio Lima | 120033_8 | 1,221 |
| 20 | COLONIA DO VINTE | Mancio Lima | 120033_70 | 1,129 |
| 10 | SAO DOMINGOS | Mancio Lima | 120033_12 | 1,038 |
| 8 | HUMAITA | Cruzeiro do Sul | 120020_76 | 1,034 |
| 13 | BAHIA | Rodrigues Alves | 120042_61 | 972 |
| 18 | BANANEIRA- P.A.D. SAO PEDRO | Rodrigues Alves | 120042_53 | 965 |
| 12 | POLO-AGROFLORESTAL | Mancio Lima | 120033_54 | 946 |
| 2 | JOSE BERNARDO | Mancio Lima | 120033_45 | 931 |
| 46 | BURITI | Mancio Lima | 120033_78 | 892 |
| 19 | MORADA NOVA | Cruzeiro do Sul | 120020_30 | 880 |
| 38 | LAGOINHA | Cruzeiro do Sul | 120020_55 | 866 |
| 27 | BURITIRANA | Cruzeiro do Sul | 120020_164 | 790 |
| 3 | CARDOSO | Mancio Lima | 120033_9 | 769 |
| 49 | GAMA (IG. GAMA) | Guajara | 130165_23 | 731 |
| 36 | IGARAPE GRANDE | Rodrigues Alves | 120042_63 | 685 |
| 11 | SANTA ROSA | Cruzeiro do Sul | 120020_84 | 581 |
| 28 | BOCA DO MOA | Cruzeiro do Sul | 120020_31 | 571 |
| 6 | COLONIA GERICO | Mancio Lima | 120033_72 | 556 |
| 9 | ASSIS BRASIL | Cruzeiro do Sul | 120020_74 | 553 |
| 14 | ALDEIA BARAO | Mancio Lima | 120033_7 | 522 |
| 37 | NOVA CINTRA | Rodrigues Alves | 120042_16 | 472 |
| 44 | TRES UNIDOS | Mancio Lima | 120033_74 | 469 |
| 17 | HAVAI | Rodrigues Alves | 120042_60 | 454 |
| 1 | URBAN AREA | Mancio Lima | 120033_111 | 442 |
| 29 | MIRITIZAL | Cruzeiro do Sul | 120020_89 | 441 |
| 56 | VILA SANTA LUZIA (BR 364) | Cruzeiro do Sul | 120020_156 | 415 |
| 32 | BELO MONTE | Mancio Lima | 120033_18 | 412 |
| 25 | SAO PEDRO | Cruzeiro do Sul | 120020_81 | 388 |
| 55 | BOM JESUS | Mancio Lima | 120033_29 | 333 |
| 23 | ALEMANHA | Cruzeiro do Sul | 120020_182 | 294 |
| 43 | C. SAO FRANCISCO - PDS SAO SALVADOR | Mancio Lima | 120033_63 | 293 |
| 58 | RAMAL 03 | Cruzeiro do Sul | 120020_96 | 276 |
| 65 | RAMAL 11 | Cruzeiro do Sul | 120020_104 | 221 |
| 52 | BOM SOSSEGO | Mancio Lima | 120033_53 | 219 |
| 54 | C. TIMBAUBA - PDS SAO SAVADOR | Mancio Lima | 120033_56 | 200 |
| 16 | DESENGANO | Rodrigues Alves | 120042_62 | 199 |
| 35 | SOCO | Mancio Lima | 120033_79 | 187 |
| 40 | C. BOA VISTA -PDS SAO SALVADOR | Mancio Lima | 120033_64 | 170 |
| 62 | RAMAL SAO FRANCISCO | Cruzeiro do Sul | 120020_217 | 135 |
| 60 | ALDEIA REPUBLICA (NUKINI) | Mancio Lima | 120033_24 | 133 |
| 63 | FLORESTA (RIO JURUA) | Guajara | 130165_34 | 120 |
| 51 | ALDEIA MEIA DUZIA | Mancio Lima | 120033_51 | 117 |
| 59 | NOVO RECREIO | Mancio Lima | 120033_21 | 117 |
| 33 | PARANA DOS MOURAS | Rodrigues Alves | 120042_76 | 115 |
| 26 | AEROPORTO VELHO | Cruzeiro do Sul | 120020_2 | 104 |
| 45 | C. SAO PEDRO - PDS SAO SALVADOR | Mancio Lima | 120033_62 | 94 |
| 64 | FORMIGUEIRO (ESTR. GAMA) | Guajara | 130165_95 | 91 |
| 57 | QUEIMADAS | Mancio Lima | 120033_73 | 82 |
| 5 | AURORA | Mancio Lima | 120033_17 | 77 |
| 50 | PORTO RICO | Mancio Lima | 120033_33 | 74 |
| 48 | ZUMIRA | Mancio Lima | 120033_83 | 71 |
| 24 | CONJUNTO SAO SALVADOR | Cruzeiro do Sul | 120020_168 | 66 |
| 41 | NOVA FLORESTA | Guajara | 130165_11 | 64 |
| 42 | PERI-PERI | Mancio Lima | 120033_22 | 61 |
| 22 | BOM JARDIM | Mancio Lima | 120033_84 | 53 |
| 31 | URBAN AREA | Rodrigues Alves | 120042_1 | 50 |
| 66 | SERRA DO MOA F/P | Mancio Lima | 120033_26 | 46 |
| 34 | LIMAO | Mancio Lima | 120033_80 | 37 |
| 53 | AQUIDABAN | Mancio Lima | 120033_16 | 21 |
| 47 | BELO HORIZONTE | Mancio Lima | 120033_75 | 9 |
| 39 | URBAN AREA | Guajara | 130165_8 | 8 |
| 30 | URBAN AREA | Cruzeiro do Sul | 120020_1 | 1 |
