# Supplementary material for: Human mobility and urban malaria risk in the main transmission hotspot of Amazonian Brazil
Source: PLoS One. 2020 Nov 25;15(11):e0242357. doi: 10.1371/journal.pone.0242357 (PMC7688137; doi:10.1371/journal.pone.0242357)
Supplement: S4 Table — (DOCX) [file pone.0242357.s007.docx]

S4 Table. Malaria cases according to most likely local of infection (either urban or rural) for all municipalities in the Amazon and selected municipalities in the Upper Juruá Valley of Brazil, 2016-2018.

|  | Malaria cases | | | % | |
| --- | --- | --- | --- | --- | --- |
| Municipality | Urban | Rural | Total | Urban | Rural |
| All municipalities in the Amazon | 67,700 | 383,479 | 451,179 | 15.0 | 85.0 |
| Mâncio Lima | 11,550 | 12,171 | 23,721 | 48.7 | 51.3 |
| Cruzeiro do Sul | 8,033 | 43,181 | 51,214 | 15.7 | 84.3 |
| Rodrigues Alves | 639 | 13,033 | 13,672 | 4.7 | 95.3 |
| Guajará | 669 | 5976 | 6645 | 10.1 | 89.9 |
