# Supplementary material for: Human mobility and urban malaria risk in the main transmission hotspot of Amazonian Brazil
Source: PLoS One. 2020 Nov 25;15(11):e0242357. doi: 10.1371/journal.pone.0242357 (PMC7688137; doi:10.1371/journal.pone.0242357)
Supplement: S6 Table — (DOCX) [file pone.0242357.s009.docx]

| S6 Table. Mixed-effects logistic regression results with determinants of urban-to-rural overall mobility (left columns) and mobility to high-risk areas (right columns), for males 16-60 years old (n = 535). | | | | | | | | | | | | |
| --- | --- | --- | --- | --- | --- | --- | --- | --- | --- | --- | --- | --- |
|  | **Overall mobility**  **(overnight (s) outside the town within the past 12 months — yes/no)** | | | | | | **Mobility to high-risk areas**  **(overnight(s) in localities with API >442 within the past 12 months — yes/no)** | | | | | |
|  | **Unadjusted model** | | | **Adjusted model** | | | **Unadjusted model** | | | **Adjusted model** | | |
|  | **OR^a^** | **(95% CI)^b^** | ***P-value*** | **OR^a^** | **(95% CI)^b^** | ***P-value*** | **OR^a^** | **(95% CI)^b^** | ***P-value*** | **OR^a^** | **(95% CI)^b^** | ***P-value*** |
| Literacy |  |  |  |  |  |  |  |  |  |  |  |  |
| Illiterate | Reference |  |  | Reference |  |  | Reference |  |  | Reference |  |  |
| Literate | 0.49 | (0.3-0.8) | 0.009 | 0.57 | (0.3-1.0) | 0.054 | 1.17 | (0.6-2.2) | 0.641 |  |  |  |
| Work status |  |  |  |  |  |  |  |  |  |  |  |  |
| Does not work | Reference |  |  | Reference |  |  | Reference |  |  | Reference |  |  |
| Formal employee | 1.52 | (0.7-3.1) | 0.258 | 1.52 | (0.8-3.0) | 0.233 | 1.73 | (0.8-3.9) | 0.179 | 1.76 | (0.8-4.0) | 0.168 |
| Informal employee | 2.82 | (1.5-5.2) | 0.001 | 2.29 | (1.3-4.0) | 0.004 | 2.59 | (1.4-4.8) | 0.003 | 2.60 | (1.4-4.9) | 0.003 |
| Employer | 0.73 | (0.0-10.9) | 0.819 | 0.54 | (0.0-6.5) | 0.628 | 2.24 | (0.2-31.9) | 0.551 | 2.19 | (0.2-31.0) | 0.561 |
| Wealth index |  |  |  |  |  |  |  |  |  |  |  |  |
| Poorest | Reference |  |  | Reference |  |  | Reference |  |  | Reference |  |  |
| Intermediate | 0.49 | (0.3-0.8) | 0.003 | 0.51 | (0.3-0.8) | 0.009 | 1.27 | (0.7-2.3) | 0.416 |  |  |  |
| Least poor | 0.61 | (0.4-1.0) | 0.038 | 0.77 | (0.5-1.3) | 0.317 | 0.89 | (0.5-1.6) | 0.698 |  |  |  |
| Cash transfer |  |  |  |  |  |  |  |  |  |  |  |  |
| No | Reference |  |  | Reference |  |  | Reference |  |  | Reference |  |  |
| Yes | 1.23 | (0.8-1.8) | 0.304 |  |  |  | 1.14 | (0.7-1.8) | 0.566 |  |  |  |
| Fishing |  |  |  |  |  |  |  |  |  |  |  |  |
| No | Reference |  |  | Reference |  |  | Reference |  |  | Reference |  |  |
| Yes | 2.01 | (1.3-3.1) | 0.001 | 1.58 | (1.0-2.4) | 0.040 | 1.62 | (1.0-2.6) | 0.049 |  |  |  |
| Second residence |  |  |  |  |  |  |  |  |  |  |  |  |
| No | Reference |  |  | Reference |  |  | Reference |  |  | Reference |  |  |
| Yes | 2.22 | (1.3-3.7) | 0.002 | 2.10 | (1.2-3.6) | 0.006 | 2.22 | (1.2-4.0) | 0.008 | 2.28 | (1.2-4.2) | 0.008 |

^a^OR= odds ratio

^b^CI= confidence interval
