# Supplementary material for: Human mobility and urban malaria risk in the main transmission hotspot of Amazonian Brazil
Source: PLoS One. 2020 Nov 25;15(11):e0242357. doi: 10.1371/journal.pone.0242357 (PMC7688137; doi:10.1371/journal.pone.0242357)
Supplement: S7 Table — (DOCX) [file pone.0242357.s010.docx]

| S7 Table. Mixed-effects negative binomial regression results with determinants of urban-to-rural overall mobility (left columns) and mobility to high-risk areas (right columns), for males 16-60 years old (n = 535). | | | | | | | | | | | |  |
| --- | --- | --- | --- | --- | --- | --- | --- | --- | --- | --- | --- | --- |
|  | **Overall mobility**  **(number of overnights outside the town within the past 12 months)** | | | | | | **Mobility to high-risk areas**  **(number of overnights in localities with an API >442 within the past 12 months)** | | | | | |
|  | **Unadjusted model** | | | **Adjusted model** | | | **Unadjusted model** | | | **Adjusted model** | | |
|  | **IRR^a^** | **(95% CI)^b^** | ***P*** | **IRR^a^** | **(95% CI)^b^** | ***P*** | **IRR^a^** | **(95% CI)^b^** | ***P*** | **IRR^a^** | **(95% CI)^b^** | ***P-value*** |
| Literacy |  |  |  |  |  |  |  |  |  |  |  |  |
| Illiterate | Reference |  |  | Reference |  |  | Reference |  |  | Reference |  |  |
| Literate | 0.50 | (0.3-1.0) | 0.054 | 0.60 | (0.3-1.2) | 0.145 | - | - | - |  |  |  |
| Work status |  |  |  |  |  |  |  |  |  |  |  |  |
| Does not work | Reference |  |  | Reference |  |  | Reference |  |  | Reference |  |  |
| Formal employee | 1.42 | (0.6-3.1) | 0.382 | 2.09 | (1.0-4.6) | 0.066 | 1.54 | (0.5-5.1) | 0.480 |  |  |  |
| Informal employee | 1.71 | (1.0-3.1) | 0.071 | 1.98 | (1.1-3.5) | 0.019 | 1.84 | (0.8-4.5) | 0.175 |  |  |  |
| Employer | 1.22 | (0.1-21.9) | 0.894 | 2.88 | (0.2-47.9) | 0.460 | 2.41 | (0.0-196.1) | 0.695 |  |  |  |
| Wealth index |  |  |  |  |  |  |  |  |  |  |  |  |
| Poorest | Reference |  |  | Reference |  |  | Reference |  |  | Reference |  |  |
| Intermediate | 0.55 | (0.3-1.0) | 0.049 | 0.60 | (0.3-1.1) | 0.092 | 0.92 | (0.37-2.29) | 0.852 |  |  |  |
| Least poor | 0.64 | (0.4-1.2) | 0.142 | 0.60 | (0.3-1.1) | 0.089 | 1.02 | (0.41-2.53) | 0.970 |  |  |  |
| Cash transfer |  |  |  |  |  |  |  |  |  |  |  |  |
| No | Reference |  |  | Reference |  |  | Reference |  |  | Reference |  |  |
| Yes | 0.24 | (-0.3-0.7) | 0.347 |  |  |  | 1.26 | (0.6-2.7) | 0.551 |  |  |  |
| Fishing |  |  |  |  |  |  |  |  |  |  |  |  |
| No | Reference |  |  | Reference |  |  | Reference |  |  | Reference |  |  |
| Yes | 1.47 | (0.9-2.5) | 0.151 |  |  |  | 1.19 | (0.5-2.6) | 0.669 |  |  |  |
| Second residence |  |  |  |  |  |  |  |  |  |  |  |  |
| No | Reference |  |  | Reference |  |  | Reference |  |  | Reference |  |  |
| Yes | 3.53 | (1.9-6.5) | <0.0001 | 3.50 | (1.9-6.5) | <0.0001 | 3.43 | (1.3-8.8) | <0.0001 | 3.43 | (1.3-8.8) | 0.011 |

^a^IRR= incidence rate ratio. Note that “incidence” here refers to the total number of overnights divided by the study duration (12 months).

^b^CI= confidence interval
