# Supplementary material for: Human mobility and urban malaria risk in the main transmission hotspot of Amazonian Brazil
Source: PLoS One. 2020 Nov 25;15(11):e0242357. doi: 10.1371/journal.pone.0242357 (PMC7688137; doi:10.1371/journal.pone.0242357)
Supplement: S8 Table — (DOCX) [file pone.0242357.s011.docx]

S8 Table. Most likely localities of origin of imported infections diagnosed and treated in the town of Mâncio Lima, 2016-2018.

| ID | Locality name | Municipality | Code (SIVEP) | Pv | Pf |
| --- | --- | --- | --- | --- | --- |
|  | Other localities |  |  | 1,199 | 336 |
| 4 | BATOQUE | Mancio Lima | 120033_8 | 635 | 231 |
| 10 | SAO DOMINGOS | Mancio Lima | 120033_12 | 550 | 219 |
| 15 | TONICO | Mancio Lima | 120033_15 | 515 | 144 |
| 7 | BRASILIA | Mancio Lima | 120033_14 | 463 | 124 |
| 12 | POLO-AGROFLORESTAL | Mancio Lima | 120033_54 | 299 | 86 |
| 20 | COLONIA DO VINTE | Mancio Lima | 120033_70 | 285 | 47 |
| 13 | BAHIA | Rodrigues Alves | 120042_61 | 247 | 81 |
| 11 | SANTA ROSA | Cruzeiro do Sul | 120020_84 | 175 | 59 |
| 2 | JOSE BERNARDO | Mancio Lima | 120033_45 | 169 | 52 |
| 17 | HAVAI | Rodrigues Alves | 120042_60 | 136 | 27 |
| 14 | ALDEIA BARAO | Mancio Lima | 120033_7 | 114 | 19 |
| 33 | PARANA DOS MOURAS | Rodrigues Alves | 120042_76 | 63 | 24 |
| 6 | COLONIA GERICO | Mancio Lima | 120033_72 | 60 | 16 |
| 54 | C. TIMBAUBA - PDS SAO SAVADOR | Mancio Lima | 120033_56 | 50 | 5 |
| 35 | SOCO | Mancio Lima | 120033_79 | 34 | 5 |
| 44 | TRES UNIDOS | Mancio Lima | 120033_74 | 32 | 1 |
| 60 | ALDEIA REPUBLICA (NUKINI) | Mancio Lima | 120033_24 | 31 | 5 |
| 52 | BOM SOSSEGO | Mancio Lima | 120033_53 | 30 | 2 |
| 16 | DESENGANO | Rodrigues Alves | 120042_62 | 30 | 12 |
| 59 | NOVO RECREIO | Mancio Lima | 120033_21 | 29 | 3 |
| 3 | CARDOSO | Mancio Lima | 120033_9 | 27 | 5 |
| 21 | PARANA DO PENTECOSTE | Mancio Lima | 120033_52 | 24 | 5 |
| 46 | BURITI | Mancio Lima | 120033_78 | 21 | 1 |
| 32 | BELO MONTE | Mancio Lima | 120033_18 | 20 | 2 |
| 9 | ASSIS BRASIL | Cruzeiro do Sul | 120020_74 | 19 | 6 |
| 45 | C. SAO PEDRO - PDS SAO SALVADOR | Mancio Lima | 120033_62 | 17 | 3 |
| 55 | BOM JESUS | Mancio Lima | 120033_29 | 16 | 1 |
| 43 | C. SAO FRANCISCO - PDS SAO SALVADOR | Mancio Lima | 120033_63 | 16 | 4 |
| 51 | ALDEIA MEIA DUZIA | Mancio Lima | 120033_51 | 12 | 4 |
| 40 | C. BOA VISTA -PDS SAO SALVADOR | Mancio Lima | 120033_64 | 10 | 4 |
| 22 | BOM JARDIM | Mancio Lima | 120033_84 | 10 | 3 |
| 18 | BANANEIRA- P.A.D. SAO PEDRO | Rodrigues Alves | 120042_53 | 9 | 2 |
| 42 | PERI-PERI | Mancio Lima | 120033_22 | 6 | 1 |
| 26 | AEROPORTO VELHO | Cruzeiro do Sul | 120020_2 | 5 | 0 |
| 34 | LIMAO | Mancio Lima | 120033_80 | 5 | 2 |
| 48 | ZUMIRA | Mancio Lima | 120033_83 | 5 | 0 |
| 53 | AQUIDABAN | Mancio Lima | 120033_16 | 4 | 0 |
| 66 | SERRA DO MOA F/P | Mancio Lima | 120033_26 | 4 | 1 |
| 57 | QUEIMADAS | Mancio Lima | 120033_73 | 4 | 1 |
| 30 | URBAN AREA | Cruzeiro do Sul | 120020_1 | 1 | 0 |
| 27 | BURITIRANA | Cruzeiro do Sul | 120020_164 | 1 | 0 |
| 25 | SAO PEDRO | Cruzeiro do Sul | 120020_81 | 1 | 1 |
| 29 | MIRITIZAL | Cruzeiro do Sul | 120020_89 | 1 | 0 |
| 5 | AURORA | Mancio Lima | 120033_17 | 1 | 0 |
| 37 | NOVA CINTRA | Rodrigues Alves | 120042_16 | 1 | 0 |
| 36 | IGARAPE GRANDE | Rodrigues Alves | 120042_63 | 1 | 0 |
| 49 | GAMA (IG. GAMA) | Guajara | 130165_23 | 1 | 0 |
| 61 | BADEJO DO MEIO (ESTR. DO GAMA) | Guajara | 130165_45 | 1 | 0 |
| 8 | HUMAITA | Cruzeiro do Sul | 120020_76 | 0 | 1 |
| Total |  |  |  | 5,389 | 1,545 |
