# Supplementary figures and images for: Human mobility and urban malaria risk in the main transmission hotspot of Amazonian Brazil
Source: PLoS One. 2020 Nov 25;15(11):e0242357. doi: 10.1371/journal.pone.0242357 (PMC7688137; doi:10.1371/journal.pone.0242357)

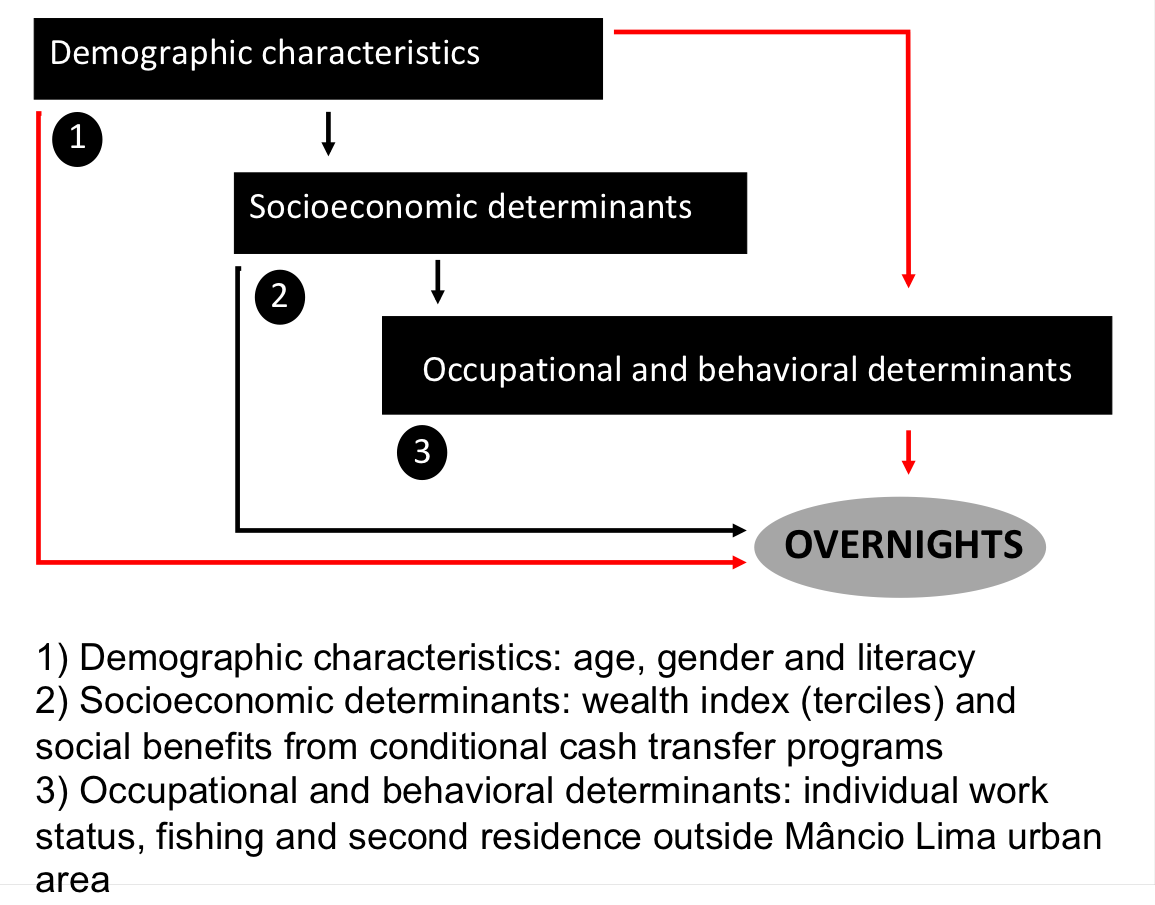

Supplement: S1 Fig — (TIFF) [file pone.0242357.s012.tiff]

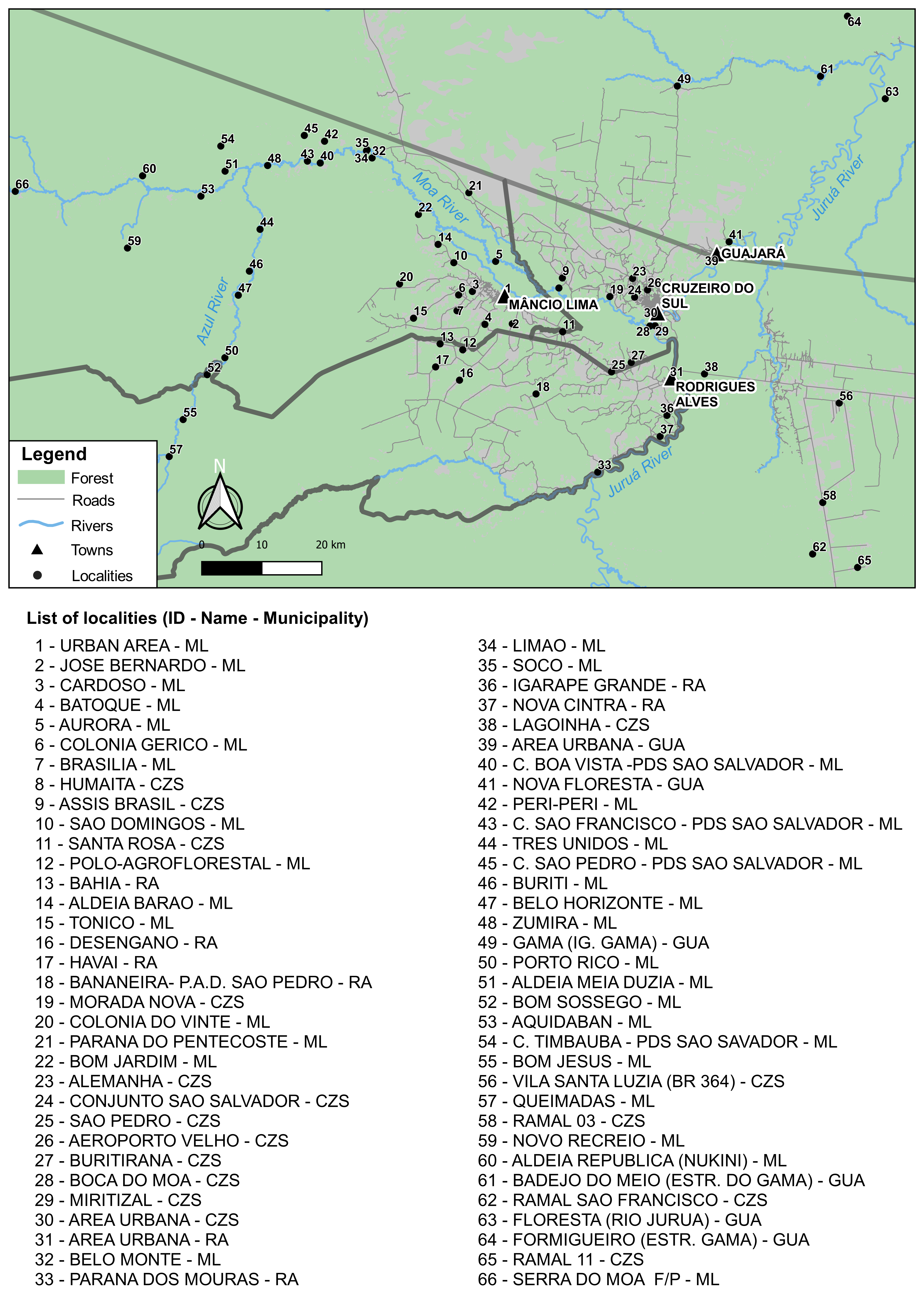

Supplement: S2 Fig — (TIF) [file pone.0242357.s013.tif]

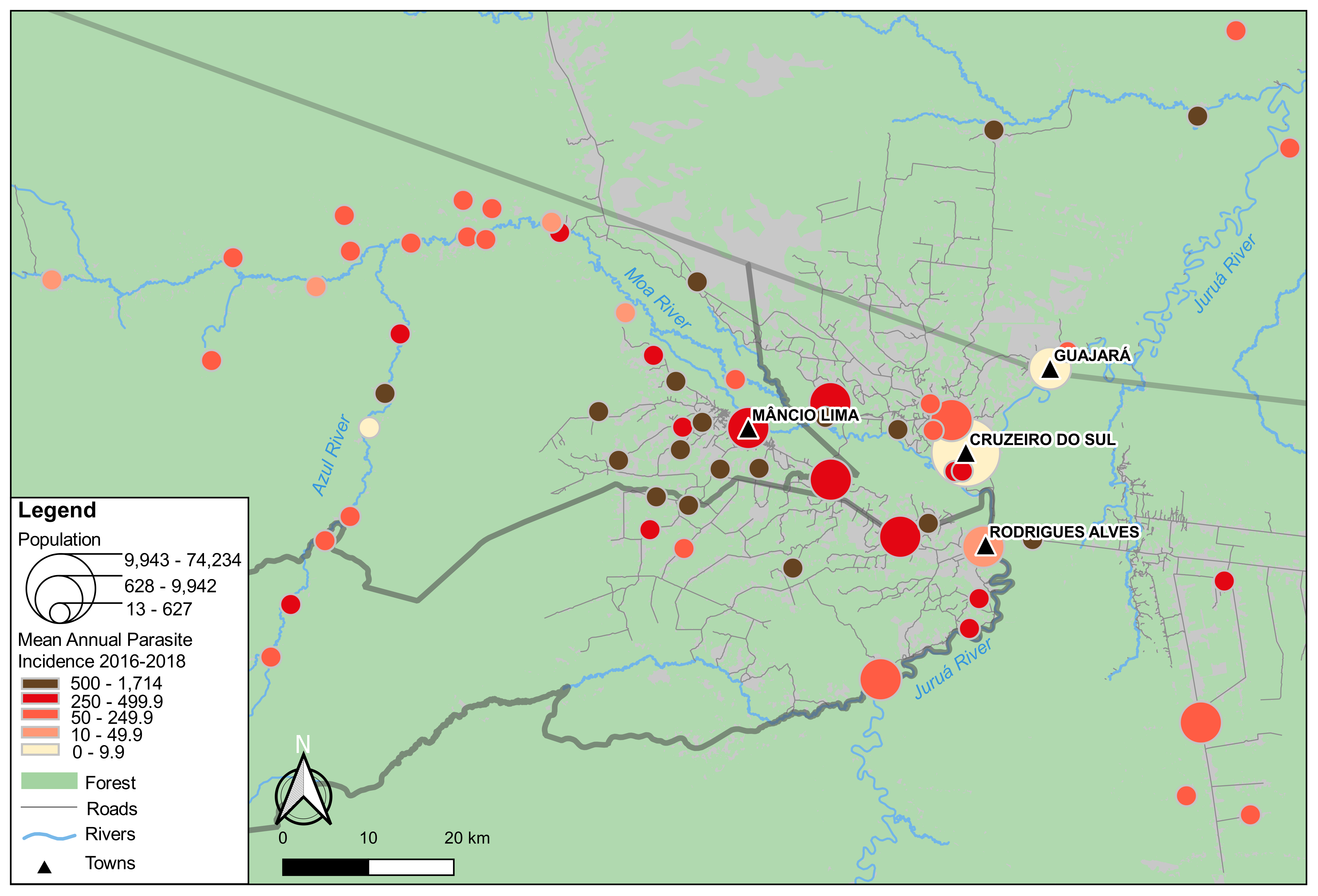

Supplement: S3 Fig — Georeferenced localities are represented by circles with size proportional to their population size and filled with tones from light yellow to dark brown that are proportional to malaria transmission intensity, using the APIs as a proxy (higher APIs in darker tones). (TIF) [file pone.0242357.s014.tif]

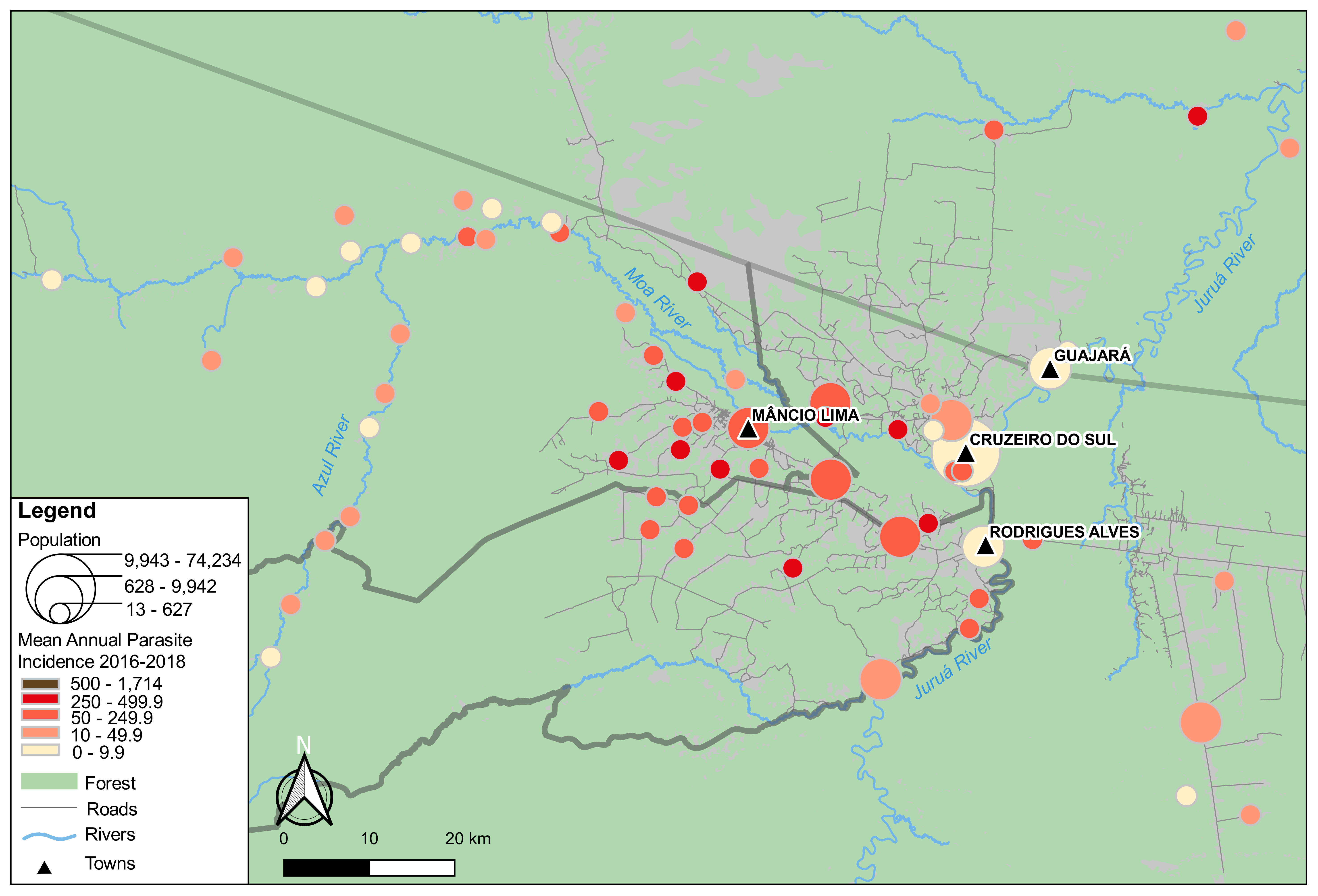

Supplement: S4 Fig — Georeferenced localities are represented by circles with size proportional to their population size and filled with tones from light yellow to dark brown that are proportional to malaria transmission intensity, using the APIs as a proxy (higher APIs in darker tones). (TIF) [file pone.0242357.s015.tif]

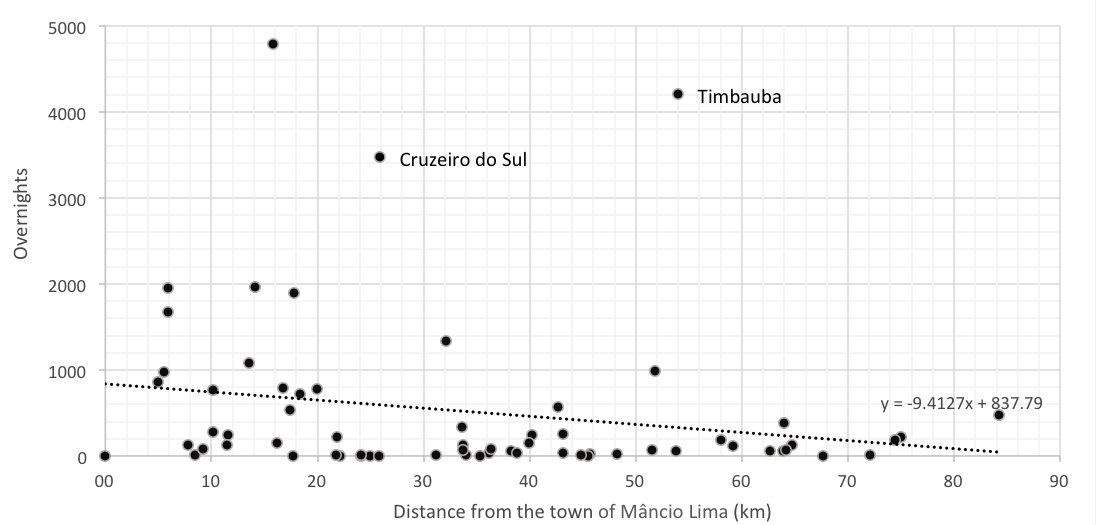

Supplement: S5 Fig — (TIFF) [file pone.0242357.s016.tiff]
